# Occupational class differences in male suicide risk in Finland from 1970 to 2019

**DOI:** 10.1093/eurpub/ckad176

**Published:** 2023-10-06

**Authors:** Simo Raittila, Anne Kouvonen, Aki Koskinen, Ari Väänänen

**Affiliations:** Faculty of Social Sciences, University of Helsinki, Helsinki, Finland; Faculty of Social Sciences, University of Helsinki, Helsinki, Finland; Centre for Public Health, Queen’s University Belfast, Belfast, UK; Finnish Institute of Occupational Health, Helsinki, Finland; Finnish Institute of Occupational Health, Helsinki, Finland

## Abstract

**Background:**

In the last few decades, suicide rates have decreased in most European countries. However, periodic changes in risk by occupational class have not been studied as much in detail.

**Methods:**

Representative cohorts of Finnish working-age men were followed for nine years on suicide mortality starting from five different census years (1970, 1980, 1990, 2000, 2010). Each cohort included between 300 970 and 332 318 men. Cox regression modelling was used to estimate hazard ratios by census year, occupational class and their interactions. Further models adjusted for age and its interactions with census year and occupational class.

**Results:**

The risk of male suicide has more than halved between 1991 and 2019. The relative hazard ratio of suicide in manual workers compared to managers and professionals was around 1.6 to 1.8 times higher. The period when the suicide risk started to decline differed by occupational class: a significant decrease compared to 1970s’ levels was seen for managers and professionals already in the 1990s and for lower non-manual employees around 10 years later (in the 2000s). Manual workers only reached the 1970s suicide risk of managers and professionals in the 2000s and 2010s.

**Conclusion:**

A delayed reduction of suicide rates among lower occupational classes suggests that the impact of social changes can occur at different speed in different population groups.

## Introduction 

Suicide mortality rate has been used as one of the key indicators to monitor population mental health.^1^ Indeed, psychological autopsy studies have found that about 90% of people who have died by suicide have had a psychiatric disorder.^2^ Due to the threshold for suicide being high, suicidal acts are considerably rarer than psychiatric disorders. At the aggregate level, higher suicide rates can thus be viewed to be an extreme indicator of increased psychosocial distress and/or vulnerability in a population—‘a peak of the iceberg’.^3^

Suicide rates have decreased in most EU countries, halving between 2000 and 2017.^4^ Also in Finland, male suicide rates have mostly declined for almost three decades. This has often been explained by developments in mental health and suicide prevention services.^5^ Earlier research has indicated that mental health services have been similarly beneficial to people in different occupational groups,^6^ although different indicators of mental health have shown varying periodical trends in risks and occupational class differences.^7^ In their meta-analysis, Milner *et al*.^8^ found support for a gradient where those in lower skilled occupations are at a higher risk of suicide. Multiple studies have found occupational class differences in suicide risk during the late 00s financial crisis, but the association with occupational class has varied in different contexts. In Australia, for example, differences widened,^9^ while in England and Wales suicides increased especially in the higher managerial and professional group, entrepreneurs and students.^10^ Certain occupations such as medical professions, agriculture work and occupations in which owning a firearm is common have been found to have higher suicide risk—due to, e.g. access to means. There is evidence for this from Finland as well.^11^

The period from the 1960s to the 2010s was characterized by considerable social transition in Finland: the rural flight of the 1960s, the build-up of the welfare state in the 1970s, the overheating of the economy in the late 1980s, followed by deep economic depression in the 1990s, and the recession of 2008–9. These macroeconomic fluctuations along with the comparatively rapid occupational shift from agricultural to industrial, service and knowledge economy jobs; as well as globalization, digitalization, increased population diversity, and changes in policies concerning issues that are associated with suicide rates (alcohol use, drug use, available medications, access to means) act as the societal context of suicide trends that can affect different socioeconomic groups and cohorts differently.^12–14^

The current study focuses on men as suicide mortality tends to be considerably rarer in women. In the EU countries, over three times as many men die by suicide than women.^13^ We are not aware of earlier studies that have investigated suicide risk by occupational class while also addressing the periodic differences in this long a timespan. Our goal in this article is to examine:

1. What kind of periods can be identified in male suicide risk in Finland from 1970 to 2019?
2. Have the occupational class differences in male suicide risk decreased during the study period?
3. Has the temporal development of suicide risk been similar in different occupational classes?

## Methods

The multi-cohort data were based on the Statistics Finland census data. Each cohort is defined by a cross-sectional sample including 33% of working-age people in Finland at the start time (census year) and followed up on mortality for 9 years. There were 5 290 296 cases in the data before limiting the study population to men (*n* = 2 639 646). The study population was limited to three occupational classes: manual workers, lower non-manual employees (lower-level employees with administrative and clerical occupations), and managers and professionals (upper-level employees with administrative, managerial, professional and related occupations*)* (total of 1 605 286 cases). This means that, for example the unemployed, self-employed and retired people, and students were not included in the analytical sample.

As table 1 displays, the highest number of suicides per 100 000 men can be seen in the 1990s cohort, expect for managers and professionals for whom the numbers were at their highest in the 1970s. The number of manual employees has strongly decreased during the study period, which can also be seen in our sample. The number of manual workers in our data was over 200 000 in the 1970s and 1980s but decreased to 160 000 by 2010. Meanwhile, there are almost twice as many managers and professionals in our 2010s’ than in our 1970s’ cohort. The change is similar in lower non-manual employees: from around 56 000 in the 1970s cohort to over 88 000 in the 2010s (table 1). This is in line with the occupational and educational shift in the Finnish workforce.^15^

**Table 1:** The number of cases and suicides by occupational class in the sample, follow-up of 9 years

	Managers and professionals			Lower non-manual employees			Manual workers		
Cohort	*n*	Suicides	Per 100 000	*n*	Suicides	Per 100 000	*n*	Suicides	Per 100 000
1970	32 397	99	3.06	55 832	166	2.97	212 741	1076	5.06
1980	49 991	108	2.16	64 658	196	3.03	209 734	1011	4.82
1990	65 853	119	1.81	76 809	243	3.16	183 515	946	5.15
2000	72 130	100	1.39	80 789	151	1.87	168 519	495	2.94
2010	82 655	67	0.81	88 284	128	1.45	161 379	338	2.09

### Variables

Occupational class represents the individuals’ status at the start of the follow-up period and is based on Statistics Finland’s 2010 Labour Force Survey Classification of Socio-Economic Groups. For older data, the earlier classifications were adapted to match this.^16^^,^^17^

The individuals in the data are deemed to have died from suicide, if their official cause of death is recorded to be suicide according to ICD-8 (1971–86), a modification of the Finnish Classification of Diseases 1987 (1987–95) or the International ICD-10 (1996–).^18^ The event—death by suicide—is measured in discrete time: at year level.

Age was measured at the start of follow-up and grouped into three age groups: 18–34, 35–49 and 50–64 years to account for different phases of working career.

### Analysis

Cause-specific Cox regression models were used to test the differences between occupational classes at different periods, as the hazard ratios from these models offer themselves to easy comparison of relative risks.^19^ The oldest decade was used as the reference for the time-period and managers and professionals for the occupational class. Interaction effects were also tested to clarify the long-term trends from changes in the age profile etc. of different cohorts.

Analyses were carried out in R version 4.2.3 and the *eha* package.^20^

## Results

As table 2 shows, over the whole studied time-period, managers and professionals always had the lowest risk of suicide. After controlling for census year (Model 1), manual workers had around 2.3 times higher and lower non-manual employees 1.4 times higher risk of suicide than managers and professionals. The decades when highest rates of suicide were observed, were the 1970s, 1980s and 1990s.

**Table 2:** Occupational class differences in male suicide risk from 1970s to 2010s in Finland

	Model 1^a^	Model 2^b^	Model 3^c^	Model 4^d^	Model 5^e^	Model 6^f^
	HR (95% CI)	HR (95% CI)	HR (95% CI)	HR (95% CI)	HR (95% CI)	HR (95% CI)
Occupational class						
Managers and professionals	1	1	1	1	1	1
Lower non-manual	1.43 (1.29, 1.60)	0.97 (0.76, 1.25)	0.98 (0.77, 1.26)	0.99 (0.77, 1.27)	0.97 (0.73, 1.29)	0.94 (0.64, 1.38)
Manual	2.30 (2.09, 2.25)	1.67 (1.36, 2.05)	1.68 (1.37, 2.07)	1.69 (1.38, 2.08)	1.82 (1.43, 2.32)	1.66 (1.20, 2.29)
Decade						
1970s	1	1	1	1	1	1
1980s	0.94 (0.87, 1.01)	0.70 (0.54, 0.92)	0.70 (0.53, 0.92)	0.75 (0.57, 1.01)	0.76 (0.57, 1.01)	0.80 (0.53, 1.23)
1990s	0.97 (0.90, 1.05)	0.59 (0.50, 0.77)	0.58 (0.44, 0.76)	0.62 (0.47, 0.82)	0.62 (0.47, 0.83)	0.53 (0.33, 0.84)
2000s	0.57 (0.52, 0.63)	0.45 (0.34, 0.59)	0.45 (0.34, 0.59)	0.45 (0.33, 0.61)	0.45 (0.33, 0.61)	0.34 (0.20, 0.58)
2010s	0.41 (0.37, 0.50)	0.26 (0.19, 0.36)	0.26 (0.19, 0.36)	0.26 (0.19, 0.37)	0.26 (0.19, 0.37)	0.21 (0.11, 0.40)
One-way interactions of period and class						
L. non-manual × 1980s		1.44 (1.03, 2.03)	1.45 (1.03, 2.04)	1.43 (1.02, 2.01)	1.42 (1.01, 2.00)	1.37 (0.81, 2.30)
L. non-manual × 1990s		1.80 (1.29, 2.51)	1.80 (1.29, 2.51)	1.79 (1.28, 2.49)	1.76 (1.26, 2.45)	1.82 (1.04, 3.17)
L. non-manual × 2000s		1.39 (0.97, 1.98)	1.38 (0.97, 1.97)	1.38 (0.96, 1.96)	1.36 (0.95, 1.94)	1.70 (0.89, 3.24)
L. non-manual × 2010s		1.84 (1.25, 2.71)	1.83 (1.25, 2.70)	1.84 (1.25, 2.71)	1.84 (1.25, 2.72)	1.91 (0.92, 3.99)
Manual × 1980s		1.35 (1.01, 1.79)	1.35 (1.02, 1.80)	1.34 (1.00, 1.78)	1.33 (1.00, 1.78)	1.24 (0.80, 1.94)
Manual × 1990s		1.72 (1.30, 2.28)	1.72 (1.30, 2.28)	1.71 (1.29, 2.26)	1.72 (1.30, 2.28)	2.09 (1.29, 3.38)
Manual × 2000s		1.28 (0.95, 1.72)	1.27 (0.94, 1.71)	1.27 (0.94, 1.71)	1.29 (0.95, 1.74)	1.79 (1.02, 3.13)
Manual × 2010s		1.56 (1.12, 2.17)	1.56 (1.12, 2.18)	1.56 (1.12, 2.18)	1.59 (1.13, 2.22)	2.09 (1.09, 4.01)

Hazard ratios (HRs) from Cox Regression with 95% confidence intervals in brackets; age effects and interaction effects reported in online-only Supplementary appendix S1.

a Model 1 includes the main effects of census year and occupational class.

b Model 2 includes the main effects and interactions of census year and occupational class.

c Model 3 additionally adjusts for age at census year.

d Model 4 additionally adjusts for interactions of census year and age.

e Model 5 additionally adjusts for interactions of occupational class and age.

f Model 6 additionally adjusts for a three-way-interaction of occupational class, census year and age.

When the interaction of occupational class and census year was taken into account in Model 2—that is, periodic differences by occupational class are assumed—a declining trend of suicide for managers and professionals was found (HR = 0.70 in the 1980s, HR = 0.59 in the 1990s, HR = 0.45 in the 2000s and HR = 0.26 in the 2010s). The statistically significant main effect between lower non-manual employees and the managerial–professional class in Model 1 (HR = 1.43) became statistically not significant in Model 2 (HR = 0.97), which suggests that this difference has varied through time.

During the study period, the difference between manual workers and the highest occupational class decreased but did not disappear (HR = 1.67 in Model 2). Hazard ratios are multiplicative, so while this main effect showed the difference in the 1970s, the total relative risk for manual employees compared to managers and professionals was 1.69 in the 1990s, for example [*occupational class* 1.67 × *decade* 0.59 × *interaction* 1.72 = 1.69].

In the decades for which the interaction effects were positive, relative suicide risks were heightened compared to managers and professionals at the same time-period. Relative risks were increased for lower non-manual employees in 1980s (HR = 1.44), 1990s (HR = 1.80), and 2010s (HR = 1.84), and manual workers in the same periods (HR = 1.35 in 1980s, HR = 1.72 in 1990s, HR = 1.56 in 2010s). In the 2000s, neither lower non-manual employees nor manual workers had a statistically significantly different risk of suicide compared to managers and professionals.

Over the whole studied time-period of almost half a decade, compared to the youngest age group, men aged 35–49 had a higher risk of suicide (HR = 1.12) when assuming that occupational class and period have affected all age groups similarly (Model 3, Supplementary appendix S1). The effects of the age adjustment on occupational class differences in suicide were negligible for all studied periods.

The model that allowed the effect of age to vary between time-periods showed that in the 1970s, men aged 35–49 had a higher risk of suicide (HR = 1.15) than those aged 18–34, and that there were no statistically different variations from this in later decades. The risk of suicide decreased for those aged 50–64 in the 1980s (HR = 0.78) compared to the 1970s (HR = 1.13) (Model 4, Supplementary appendix S1).

Model 4 assumed that the time-variable age effects are similar in different occupational classes. Model 5 relaxed this assumption. Main results did not change meaningfully, but the overall hazard ratio for manual employees increased to 1.82 (Model 5). The older men’s slightly lower suicide risk in the 1980s (HR = 0.78) was only found for managers and professionals (Model 5, Supplementary appendix S1).

Model 6 allowed the exploration of the most nuanced differences. In the model, there was no fixed difference between the suicide risk of lower non-manual employees when compared to managers and professionals. This suggests that these occupational class differences are related more to the varying period effects (the effect has not been fixed for 50 years). Manual employees’ higher risk (HR = 1.66) persisted in the model.

Two-way interaction effects of period and occupational class differed from Model 5 with lower non-manual employees having statistically significant hazard ratios compared to the reference group only in the 1990s (HR = 1.82) and manual employees in the 1990s (HR = 2.09), 2000s (HR = 1.79) and 2010s (HR = 2.09). The decade-to-decade period effect persisted (Model 6, Supplementary appendix S1).

The age effect and its interactions with decade and occupational class were no longer statistically significant. This suggests more complex time-variable three-way interactions and age effects compared to the average of each occupational class in each period. The hazards from the three-way interaction have to be understood as exceptions to the main effect and other interaction effects. Such exceptions were not identified at the decade level with our age groupings.

**Figure 1:**
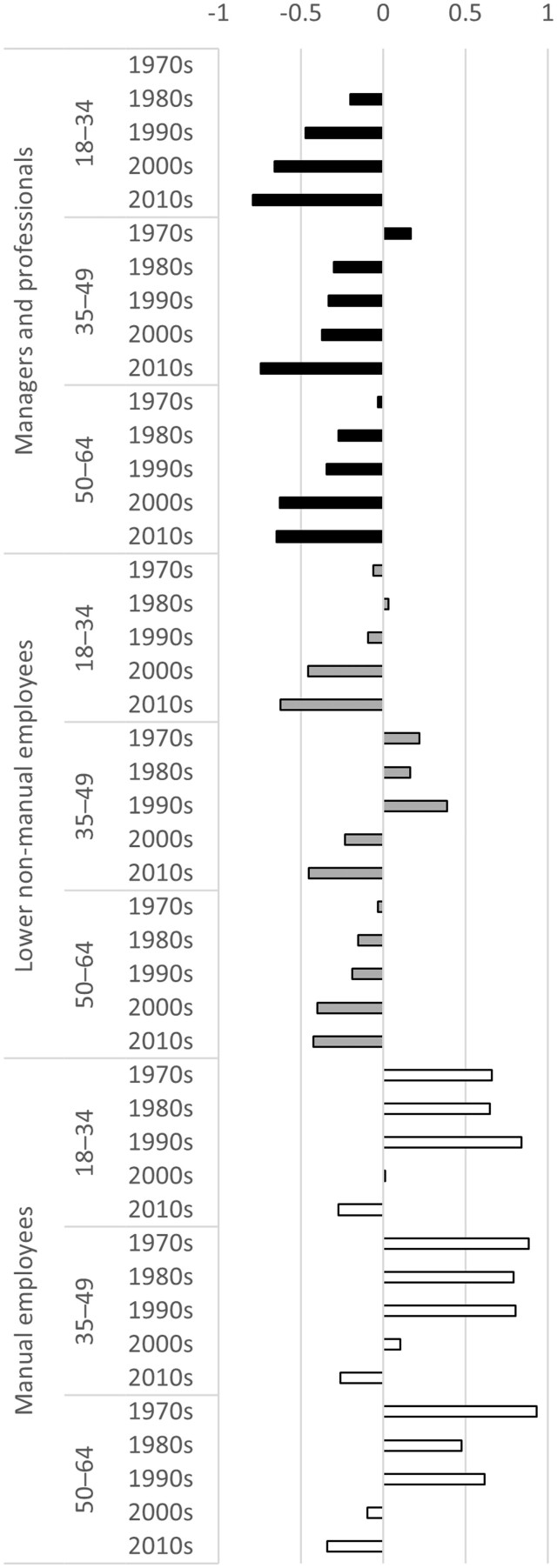
Hazard ratio difference to 18- to 34-year-old managers and professionals in the 1970s (reference group, value 0), multiplied from hazard ratios of Model 6

Multiplied hazard ratios from Model 6 are visualized in figure 1. A rapid decrease in suicide risk is seen decade-to-decade in managers and professionals of all ages. For lower non-manual employees, the change is only observable from the 2000s onwards, and the decrease seems to be stronger in the youngest age group. Manual workers only reached the 1970s suicide risk of managers and professionals in the 2000s and 2010s.

**Figure 2:**
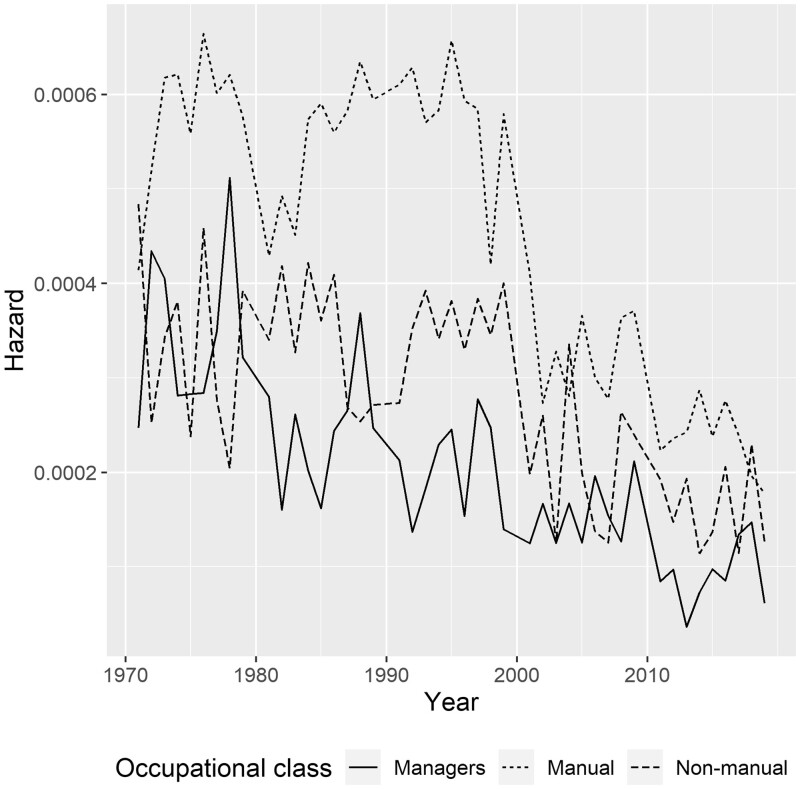
Hazard of suicide by year in 5 followed cohorts (each cohort modelled separately; results combined)

Year-to-year hazards for each cohort were also modelled separately to visualize the yearly risk of death by suicide (figure 2). A declining trend was clear for managers and professionals from 1971 to 2019, with singular peaks in late 1970s and 1980s. There were already signs of a similar decrease in hazard in lower non-manual employees from 1987 to 1991, but their suicide risk rose back to earlier levels during the 1990s. This affected mostly lower non-manual employees aged 35–49 (figure 1). Lower non-manual employees reached the managers’ and professionals’ level in the 2000s. There was a considerable decrease in suicide risk in manual workers in early 1980s, but their risk mostly remained high for the whole of 1970–2000s, only starting to decline sharply in the late 1990s and the early 2000s.

## Discussion

Suicides of working-age men in Finland have about halved since the early 1990s. The suicide risk decreased first in the managerial and professional class, followed by lower non-manual employees and finally among manual workers. Manual workers did not reach the risk levels of managers and professionals in 1970s before the turn of the millennia.

From the perspective of societal change and the equality of population groups, the time dependent nature of a transition to lower suicide risk by occupational class is an important finding. Based on our finding, there seems to be at least three distinct periods of occupational class differences in male suicide risk that can be recognized in Finland:

1. The period of high suicide risk and high occupational class differences (around 1970–96)
2. The period of equalization of suicide risk (around 1996–2003, when the relative risk decreased sharply in both non-manual and manual employees)
3. The period of overall lower suicide risk (2003–19)

Between 1970 and 2019, the male suicide risk decreased in all socioeconomic groups in Finland. However, our results show clear differences in the historical development of male suicide risk in the working-age population. The relative risks persist but are less of an issue due to the absolute decrease in male suicide and occupational shift: there are both fewer manual workers, and fewer of them die by suicide. In 1970, manual workers made up 45% of the workforce.^21^ By 2021, this share had decreased to 35%.^22^

Individual, shock-like years can be found in the visualizations but were not confirmed by the models. Our results suggest that the 1990s depression affected lower non-manual employees comparatively even more and for a longer time-period than manual workers. However, even though they can be argued to have been hit relatively harder by the economic depression, manual workers still had the highest risk of suicide even during that period.

According to our results, the suicide risk for these two groups remained high (manual workers) or increased (non-manual employees) during the 1990s. Meanwhile, the suicide risk of managers and professionals continued to decrease. There is some disagreement in earlier literature on whether suicides increase before or during economic recessions.^10^^,^^13^^,^^23^^,^^24^ Our results do not provide strong evidence for either association, but show that the effect and timing can differ by occupational class.

Multiple mechanisms might be at work simultaneously: the better situation of managers and professionals could be at least partly explained by, for example, better access to care, more efficient use and early adoption of psychotropic medications, and health behaviours such as less heavy drinking or not drinking due to using antidepressants.^25–27^

Earlier research of the effect of the widespread introduction of SSRIs (selective serotonin uptake inhibitors) on male suicide in the 1990s in Nordic countries is mixed and in many countries suicide rates had started to decline already before the introduction of these new medications. In Finland though, the introduction coincided with the start of suicide prevention programme and the starting decline in suicide risk, as seen in our visualizations. According to Reseland *et al*.,^28^ the prevention programmes in Norway (1992) and Sweden (1995) did not have a similar temporal association in their respective populations. It is not straightforward to put a starting year for all projects though. In Norway, 1992 saw the introduction of a phone helpline, but different sources consider a suicide prevention strategy to have properly started only either in 1994 or 1995.^29^^,^^30^

The implementation phase of the first of its kind national suicide prevention programme in Finland started in 1992 but was preceded by a research phase in 1986–91. This programme cannot thus be the main driver of the decrease in suicides of managers and professionals, which was evident already from the 1970s.

There were also earlier major mental health research programmes in Finland: UKKI in the 1970s and the Mini Finland study in 1977–81.^31^ These might have affected the perception of mental distress in different occupational classes to a different degree and at different times and explain a part of the earlier changes in the managerial and professional class. Before the 1990s, psychiatric epidemiology primarily emphasized social psychiatry and service development. Concurrently, research has identified the influential role of labour unions in advancing mental well-being in the workplace. However, in the 1990s, a shift towards individualism occurred in psychiatric epidemiology, with a greater emphasis on biomedical well-being. The professional discourses and occupational debates also began to advocate for more personal responsibility of the employees’ own well-being.^31^^,^^32^

Meanwhile, the recession of 2008–9 shows in our visualization only as a small rise of suicide risk or a pause in the decreasing trend for couple of years in all studied occupational classes. After this, the decline of risk of suicide continued. This is in line with earlier research on ‘the Great Recession’.^33^^,^^34^

Suicides still account for about 10% of the socioeconomic differences in life expectancies in Finland.^35^ Our study adds to the evidence that even with the welcome decrease in male suicide risk in Finland, a risk difference between men in manual jobs and those in non-manual jobs remains.

### Strengths and limitations

The large and comprehensive data available in Finnish administrative registers is a major strength of this study. Almost half a century’s time could be analysed quantitatively in a single study with representative samples of around a third of Finnish working-age male population for each decade.

A major limitation is that the currently available data did not allow for an analysis of how changes in a person’s life-course affected the risk of suicide. Suicide is known to be related to adverse life changes such as losing a job or a loved one or one’s own degrading health,^10^^,^^24^^,^^36^ as well as longer-term social isolation and feelings of loneliness.^37^ Such situations were beyond the scope of the current study.

Thus, our study describes the comparative risk of suicide, but it does not explain the pathways that lead to issues of mental health and suicidal action; the ‘causes-of-causes’,^38^ through which the socioeconomic differences emerge. One such major limitation is that we do not take into account earlier history of psychiatric disorders. Including such measures in later studies would allow for a more in-depth study of what might cause the periodic-socioeconomic differences in suicide risk.

Suicide is a comparatively rare phenomenon, and therefore even large administrative data are not necessarily enough to statistically test the differences year by year. This limits the interpretation of the data as it is hard to associate changes in suicide rates even to aggregate-level events or clear times of policy changes.

We only focused on men in certain occupational classes. Our study did not include women, students, self-employed or the retired people, or the unemployed. More research on women and those outside the labour market is needed. Our study has nevertheless provided novel information about the development of suicide risks in the main occupational classes in men.

## Conclusion

Our study showed that the decrease in male suicide risk has occurred at different times for different occupational classes. In relative terms, occupational class differences persist. Such differences should continue to be monitored and services tailored to groups and occupations especially at risk,^8^ although earlier research has also suggested that, in line with the Rose theorem, suicide prevention should be targeted not only to high-risk groups but at population at large.^39^ More attention should be paid on the interaction between societal context and mental health^12^; and the reasons behind the differential ‘timelines’ should be investigated further to increase our understanding of the societal context of mental health.

## Supplementary Material

ckad176_Supplementary_Data

## Data Availability

Data in this study were used under its’ license approved by Statistics Finland, the Social Insurance Institution of Finland, and Finnish Centre for Pensions. They are not publicly available. Currently, the Finnish Social and Health Data Permit Authority (Findata) coordinates the permissions for similar datasets, and they are available to researchers under permission (https://findata.fi/en/).

## References

[1] Patel V , Saxena S, Lund CL, et al The Lancet Commission on global mental health and sustainable development. Lancet 2018;392:1553–98. 30314863 10.1016/S0140-6736(18)31612-X

[2] Cavanagh JTO , Carson AJ, Sharpe M, Lawrie SM. Psychological autopsy studies of suicide: a systematic review. Psychol Med 2003;33:395–405. 12701661 10.1017/s0033291702006943

[3] Geulayov G , Casey D, McDonald KC, et al Incidence of suicide, hospital-presenting non-fatal self-harm, and community-occurring non-fatal self-harm in adolescents in England (the iceberg model of self-harm): a retrospective study. Lancet Psychiatry 2018;5:167–74. 29246453 10.1016/S2215-0366(17)30478-9

[4] OECD/European Union. Health at a Glance: Europe 2020: State of Health in the EU Cycle. Health at a Glance 2020. Paris: OECD Publishing, 2020.

[5] Mäki N. Not in all Walks of Life?: Social Differences in Suicide Mortality [dissertation]. Helsinki: Helsinki University Print, 2010.

[6] Varje P , Kokkinen L, Kouvonen A, et al Occupational groups and main causes of hospitalization. J Occup Environ Med 2014;56:886–91. 25099417 10.1097/JOM.0000000000000192

[7] Varje P , Kouvonen A, Kokkinen L, et al Occupational class and the changing patterns of hospitalization for affective and neurotic disorders: a nationwide register-based study of the Finnish working-age population, 1976–2010. Soc Psychiatry Psychiatr Epidemiol 2018;53:131–8. 29236136 10.1007/s00127-017-1472-z

[8] Milner A , Spittal MJ, Pirkis J, LaMontagne AD. Suicide by occupation: systematic review and meta-analysis. Br J Psychiatry 2013;203:409–16. 24297788 10.1192/bjp.bp.113.128405

[9] Milner AJ , Niven H, LaMontagne AD. Occupational class differences in suicide: evidence of changes over time and during the global financial crisis in Australia. BMC Psychiatry 2015;15:223. 26391772 10.1186/s12888-015-0608-5 PMC4578370

[10] Coope C , Gunnell D, Hollingworth W, et al Suicide and the 2008 economic recession: Who is most at risk? Trends in suicide rates in England and Wales 2001–2011. Soc Sci Med 2014;117:76–85. 25054280 10.1016/j.socscimed.2014.07.024 PMC4151136

[11] Rinne H , Parkkinen M, Shemeikka R, et al Kuolleisuus ja työkyvyttömyyseläkkeelle siirtyminen palkansaajilla ammateittain Suomessa 2001–2015 (Mortality and Transitions to Disability Pension in Wage Earners by Occupation in Finland 2001–2015). Helsinki, Finland: Rehabilitation Foundation; 2018 Kuntoutussäätiön tutkimuksia 90/2018.

[12] Chandler A. Socioeconomic inequalities of suicide: sociological and psychological intersections. Eur J Soc Theory 2020;23:33–51.

[13] Mathieu S , Treloar A, Hawgood J, et al The role of unemployment, financial hardship, and economic recession on suicidal behaviors and interventions to mitigate their impact: a review. Front Public Health 2022;10:907052. 35875017 10.3389/fpubh.2022.907052 PMC9298506

[14] Ohberg A , Lonnqvist J, Sarna S, et al Trends and availability of suicide methods in Finland: proposals for restrictive measures. Br J Psychiatry 1995;166:35–43. 7894873 10.1192/bjp.166.1.35

[15] Kerr S , Maczulskij T, Maliranta M. Within and between firm trends in job polarization: the roles of globalization and technology. J Econ Geogr 2020;20:1003–39.

[16] Luokitus: Classification of Occupations 2010 | Tilastokeskus. Available at: https://www.stat.fi/en/luokitukset/ammatti/ (20 March 2023, date last accessed).

[17] Tilastokeskus - The Classification of Socio-Economic Groups in the Labour Force Survey. Available at: https://www.stat.fi/til/tyti/tyti_2012-03-09_uut_001_en.html (6 September 2021, date last accessed).

[18] Tilastokeskus - The Key Between the Cause of Death Statistics’ 54-Group Short List and Classifications of Diseases. Available at: https://www.stat.fi/til/ksyyt/ksyyt_2021-10-29_luo_001_en.html (30 April 2023, date last accessed).

[19] Andersen PK , Geskus RB, de witte T, Putter H. Competing risks in epidemiology: possibilities and pitfalls. Int J Epidemiol 2012;41:861–70. 22253319 10.1093/ije/dyr213 PMC3396320

[20] Broström G. Event History Analysis • eha: Available at: http://ehar.se/r/eha/index.html (7 September 2020, date last accessed).

[21] Toimihenkilöiden määrä kaksinkertaistunut 40 vuodessa | Tilastokeskus (The amount of managers, professionals and lower non-manual employees has doubled in 40 years | Statistics Finland): Available at: https://www.stat.fi/tup/vl2010/art_2012-09-11_001.html (30 April 2023, date last accessed).

[22] Työllisten ja työvoiman määrä kasvoi, työikäisen väestön määrä kääntyi laskuun vuonna 2021—Tilastokeskus (The amount of employed and workforce increased, the amount of working-age population decreased in the year 2021 | Statistics Finland): Available at: https://www.stat.fi/julkaisu/cl2yinm5hzj1l0dw2f2b376he (30 April 2023, date last accessed ).

[23] Fountoulakis KN , Koupidis SA, Siamouli M, et al Suicide, recession, and unemployment. Lancet 2013;381:721–2. 10.1016/S0140-6736(13)60573-5 23472911

[24] Fountoulakis KN , Kawohl W, Theodorakis PN, et al Relationship of suicide rates to economic variables in Europe: 2000-2011. Br J Psychiatry 2014;205:486–96. 25359926 10.1192/bjp.bp.114.147454

[25] Halonen JI , Koskinen A, Kouvonen A, et al Distinctive use of newer and older antidepressants in major geographical areas: a nationally representative register-based study. J Affect Disord 2018;229:358–63. 29331694 10.1016/j.jad.2017.12.102

[26] Halonen JI , Koskinen A, Varje P, et al Mental health by gender-specific occupational groups: profiles, risks and dominance of predictors. J Affect Disord 2018;238:311–6. 29902735 10.1016/j.jad.2018.06.007

[27] Moustgaard H , Joutsenniemi K, Myrskylä M, Martikainen P. Antidepressant sales and the risk for alcohol-related and non-alcohol-related suicide in Finland—an individual-level population study. PLoS One 2014;9:e98405. 24892560 10.1371/journal.pone.0098405 PMC4043885

[28] Reseland S , Bray I, Gunnell D. Relationship between antidepressant sales and secular trends in suicide rates in the Nordic countries. Br J Psychiatry 2006;188:354–8. 16582062 10.1192/bjp.188.4.354

[29] Matsubayashi T , Ueda M. The effect of national suicide prevention programs on suicide rates in 21 OECD nations. Soc Sci Med 2011;73:1395–400. 21940085 10.1016/j.socscimed.2011.08.022

[30] Lewitzka U , Sauer C, Bauer M, Felber W. Are national suicide prevention programs effective? A comparison of 4 verum and 4 control countries over 30 years. BMC Psychiatry 2019;19:158. 31122215 10.1186/s12888-019-2147-y PMC6533665

[31] Myllykangas M , Parhi K. The history of psychiatric epidemiology in Finland: from national needs to international arenas, 1900s–1990s. Bull Hist Med 2023;97:320–49. 10.1353/bhm.2023.a905733 38588249

[32] Kuokkanen A , Varje P, Väänänen A. Struggle over employees psychological well-being. The politization and depolitization of the debate on employee mental health in the Finnish insurance sector. Manage Organ Hist 2020;15:252–72.

[33] Reeves A , McKee M, Stuckler D. Economic suicides in the Great Recession in Europe and North America. Br J Psychiatry 2014;205:246–7. 24925987 10.1192/bjp.bp.114.144766

[34] Chang SS , Stuckler D, Yip P, Gunnell D. Impact of 2008 global economic crisis on suicide: time trend study in 54 countries. 2013;347:f5239. 10.1136/bmj.f5239 PMC3776046 24046155

[35] Partonen T. A new national suicide prevention programme in Finland. Psychiatria Fennica 2020;51:10–5.

[36] Conejero I , Olié E, Courtet P, Calati R. Suicide in older adults: current perspectives. Clin Interv Aging 2018;13:691–9. 29719381 10.2147/CIA.S130670 PMC5916258

[37] Calati R , Ferrari C, Brittner M, et al Suicidal thoughts and behaviors and social isolation: a narrative review of the literature. J Affect Disord 2019;245:653–67. 30445391 10.1016/j.jad.2018.11.022

[38] Marmot M. Social determinants of health inequalities. Lancet 2005;365:1099–104. 15781105 10.1016/S0140-6736(05)71146-6

[39] Yip PSF , So BK, Kawachi I, et al A Markov chain model for studying suicide dynamics: an illustration of the Rose theorem. BMC Public Health 2014;14:625. 24948330 10.1186/1471-2458-14-625 PMC4082176

