# Supplementary material for: Occupational class differences in male suicide risk in Finland from 1970 to 2019
Source: Eur J Public Health. 2023 Oct 6;33(6):1014–9. doi: 10.1093/eurpub/ckad176 (PMC10710325; doi:10.1093/eurpub/ckad176)
Supplement: ckad176_Supplementary_Data [file ckad176_supplementary_data.docx]

# Appendix 1: Table 2 with omitted variables (Models 3–6)

|  | Model 3 | Model 4 | Model 5 | Model 6 |
| --- | --- | --- | --- | --- |
|  | HR (95 % CI) | HR (95 % CI) | HR (95 % CI) | HR (95 % CI) |
| Occupational class |  |  |  |  |
| Managers & professionals | 1 | 1 | 1 | 1 |
| Lower non-manual | 0.98 (0.77, 1.26) | 0.99 (0.77, 1.27) | 0.97 (0.73, 1.29) | 0.94 (0.64, 1.38) |
| Manual | 1.68 (1.37, 2.07) | 1.69 (1.38, 2.08) | 1.82 (1.43, 2.32) | 1.66 (1.20, 2.29) |
| Decade |  |  |  |  |
| 1970s | 1 | 1 | 1 | 1 |
| 1980s | 0.70 (0.53, 0.92) | 0.75 (0.57, 1.01) | 0.76 (0.57, 1.01) | 0.80 (0.53, 1.23) |
| 1990s | 0.58 (0.44, 0.76) | 0.62 (0.47, 0.82) | 0.62 (0.47, 0.83) | 0.53 (0.33, 0.84) |
| 2000s | 0.45 (0.34, 0.59) | 0.45 (0.33, 0.61) | 0.45 (0.33, 0.61) | 0.34 (0.20, 0.58) |
| 2010s | 0.26 (0.19, 0.36) | 0.26 (0.19, 0.37) | 0.26 (0.19, 0.37) | 0.21 (0.11, 0.40) |
| Class × Decade |  |  |  |  |
| L. non-manual × 1980s | 1.45 (1.03, 2.04) | 1.43 (1.02, 2.01) | 1.42 (1.01, 2.00) | 1.37 (0.81, 2.30) |
| L. non-manual × 1990s | 1.80 (1.29, 2.51) | 1.79 (1.28, 2.49) | 1.76 (1.26, 2.45) | 1.82 (1.04, 3.17) |
| L. non-manual × 2000s | 1.38 (0.97, 1.97) | 1.38 (0.96, 1.96) | 1.36 (0.95, 1.94) | 1.70 (0.89, 3.24) |
| L. non-manual × 2010s | 1.83 (1.25, 2.70) | 1.84 (1.25, 2.71) | 1.84 (1.25, 2.72) | 1.91 (0.92, 3.99) |
| Manual × 1980s | 1.35 (1.02, 1.80) | 1.34 (1.00, 1.78) | 1.33 (1.00, 1.78) | 1.24 (0.80, 1.94) |
| Manual × 1990s | 1.72 (1.30, 2.28) | 1.71 (1.29, 2.26) | 1.72 (1.30, 2.28) | 2.09 (1.29, 3.38) |
| Manual × 2000s | 1.27 (0.94, 1.71) | 1.27 (0.94, 1.71) | 1.29 (0.95, 1.74) | 1.79 (1.02, 3.13) |
| Manual × 2010s | 1.56 (1.12, 2.18) | 1.56 (1.12, 2.18) | 1.59 (1.13, 2.22) | 2.09 (1.09, 4.01) |
| Age |  |  |  |  |
| 18–34 | 1 | 1 | 1 | 1 |
| 35–49 | 1.12 (1.05, 1.19) | 1.15 (1.02, 1.30) | 1.25 (0.99, 1.58) | 1.17 (0.76, 1.80) |
| 50– | 0.98 (0.91, 1.06) | 1.13 (0.98, 1.32) | 1.29 (0.97, 1.73) | 0.97 (0.54, 1.73) |
| Decade × Age |  |  |  |  |
| 1980s × 35–49 |  | 0.92 (0.78, 1.09) | 0.92 (0.77, 1.09) | 0.75 (0.41, 1.35) |
| 1990s × 35–49 |  | 0.94 (0.80, 1.11) | 0.93 (0.78, 1.10) | 1.08 (0.59, 1.98) |
| 2000s × 35–49 |  | 1.06 (0.86, 1.29) | 1.03 (0.84, 1.26) | 1.58 (0.81, 3.08) |
| 2010s × 35–49 |  | 0.97 (0.77, 1.22) | 0.94 (0.74, 1.18) | 1.04 (0.47, 2.30) |
| 1980s × 50– |  | 0.78 (0.63, 0.98) | 0.78 (0.62, 0.98) | 0.94 (0.42, 2.08) |
| 1990s × 50– |  | 0.80 (0.64, 1.00) | 0.79 (0.63, 1.00) | 1.28 (0.58, 2.82) |
| 2000s × 50– |  | 0.83 (0.64, 1.07) | 0.82 (0.63, 1.06) | 1.13 (0.49, 2.63) |
| 2010s × 50– |  | 0.97 (0.75, 1.26) | 0.95 (0.73, 1.24) | 1.73 (0.72, 4.17) |
| Class × Age |  |  |  |  |
| Non-manual × 35–49 |  |  | 1.12 (0.87, 1.44) | 1.11 (0.64, 1.91) |
| Non-manual × 50– |  |  | 0.94 (0.68, 1.29) | 1.06 (0.51, 2.22) |
| Manual × 35–49 |  |  | 0.89 (0.71, 1.10) | 0.97 (0.62, 1.52) |
| Manual × 50– |  |  | 0.86 (0.66, 1.13) | 1.20 (0.66, 2.19) |
| Class × Decade × Age |  |  |  |  |
| L. non-manual × 1980s × 35–49 |  |  |  | 1.16 (0.55, 2.44) |
| L. non-manual × 1990s × 35–49 |  |  |  | 1.09 (0.52, 2.28) |
| L. non-manual × 2000s × 35–49 |  |  |  | 0.69 (0.30, 1.58) |
| L. non-manual × 2010s × 35–49 |  |  |  | 1.08 (0.42, 2.80) |
| Manual × 1980s × 35–49 |  |  |  | 1.28 (0.69, 2.40) |
| Manual × 1990s × 35–49 |  |  |  | 0.80 (0.43, 1.51) |
| Manual × 2000s × 35–49 |  |  |  | 0.61 (0.30, 1.23) |
| Manual × 2010s × 35–49 |  |  |  | 0.86 (0.37, 2.00) |
| L. non-manual × 1980s × 50– |  |  |  | 0.85 (0.31, 2.36) |
| L. non-manual × 1990s × 50– |  |  |  | 0.68 (0.25, 1.87) |
| L. non-manual × 2000s × 50– |  |  |  | 0.95 (0.33, 2.73) |
| L. non-manual × 2010s × 50– |  |  |  | 0.86 (0.29, 2.55) |
| Manual × 1980s × 50– |  |  |  | 0.82 (0.36, 1.89) |
| Manual × 1990s × 50– |  |  |  | 0.59 (0.26, 1.36) |
| Manual × 2000s × 50– |  |  |  | 0.68 (0.28, 1.66) |
| Manual × 2010s × 50– |  |  |  | 0.45 (0.18, 1.14) |
